# Analysis of the Effects of Known Sleep-Support Supplements in Relation to Life Habits, Sleep Conditions, and Sleep Problems

**DOI:** 10.3390/nu15102377

**Published:** 2023-05-19

**Authors:** Fuminori Imafuku, Kazuya Yamamoto, Eiji Tanaka, Ryo Aoki, Seiji Nishino

**Affiliations:** 1 Ezaki Glico Co., Ltd., Osaka 555-8502, Japan; kazuya.yamamoto@glico.com (K.Y.); eiji.tanaka@glico.com (E.T.); ryo.aoki@glico.com (R.A.); 2 School of Medicine, Stanford University, Palo Alto, CA 94305, USA; nishino@stanford.edu

**Keywords:** supplement, personalization, sleep

## Abstract

Sleep is a crucial component of health, and insomnia is among the most common and vexing of life-habit-related disorders. While dietary sleep-support supplements can improve sleep, choosing an effective dietary supplement can be challenging for users due to the wide variety of options available and the varying effects experienced by different individuals. In this study, to identify new criteria for estimating the effects of dietary supplements, we examined the relationships among the dietary supplements, the pre-conditions (PCs; defined as the life habits and sleep conditions before supplementation), and the sleep problems of subjects before supplementation. An open, randomized, cross-over intervention trial enrolling 160 subjects was conducted to test the efficacy of each dietary supplement (Analysis 1) and the relationships among dietary supplements, the PCs, and sleep problems (Analysis 2). To this end, l-theanine (200 mg/day), γ-aminobutyric acid (GABA) (111.1 mg/day), *Apocynum venetum* leaf extract (AVLE) (50 mg/day), and l-serine (300 mg/day) were administered to subjects. Before the first intervention period, life habits and sleep conditions were surveyed to identify each subject’s PCs. For each combination of supplements and sleep problems, PCs were compared between subjects whose sleep problems were improved and subjects whose sleep problems were not improved via supplementation. All the tested supplements were found to ameliorate sleep problems significantly (Analysis 1). In Analysis 2, the PCs specific to improved subjects were found to differ depending on the dietary supplements and sleep problems. In addition, subjects who consumed dairy products often showed improvement in their sleep problems with all the tested supplements. This study suggests the possibility of personalizing sleep-support supplementation based on personal life habits, sleep conditions, and sleep problems, in addition to the known efficacy of dietary supplements.

## 1. Introduction

Insomnia is one of the most prevalent and vexing problems in modern society, with a worldwide prevalence of 20%, and it is known to cause lifestyle-related diseases such as hypertension, type 2 diabetes mellitus, and depression [1,2]. Insomnia is categorized according to the sleep problems that cause it, such as difficulty in falling asleep and interrupted sleep. Many solutions have been proposed to ease the various sleep problems, including pharmacotherapy, cognitive behavior therapy, etc. [2]. Pharmacotherapy is the most prevalent solution at present because of its good clinical effectiveness, but it often causes adverse effects such as dependency, memory loss, and behavioral problems [3].

In parallel with classical pharmacotherapy, many dietary sleep-support supplements have been introduced and proven to be effective to decrease sleep problems. For example, it has been reported that l-theanine is effective for refreshing postmenopausal women in the morning [4], γ-aminobutyric acid (GABA) shortens sleep latency, the *Apocynum venetum* leaf extract (AVLE) shortens sleep latency and induces deep sleep [5], and l-serine decreases the frequency of awakenings [6]. Some of these supplements are superior to pharmacotherapy in their side-effect profiles and accessibility. However, it can be difficult for users to select an effective dietary supplement due to the wide range of choices and the differential effects among users. Therefore, to select an effective dietary supplement for each user, practical criteria for estimating the effect of dietary supplements on each individual are necessary. Hence, this study focused on the life habits and sleep conditions of individual subjects before supplementation (pre-conditions: PCs); for example, the frequency of vegetable consumption per week, the frequency of the occurrence of interrupted sleep the day before supplementation, etc. Some previous reports also showed that specific dietary supplements were effective for subjects with specific PCs [6]. However, only limited PCs and dietary supplements were assessed, and there has been no comprehensive report researching the relationships among dietary supplements, sleep problems, and PCs. Therefore, this study aimed to clarify the wide-ranging relationships among dietary supplements, sleep problems, and PCs. To examine these, the PCs of the improved group were compared with those of the non-improved group for all 25 combinations of 5 sleep problems and 5 supplements (l-theanine, GABA, AVLE, l-serine, and placebo).

## 2. Materials and Methods

### 2.1. Clinical Study

**Figure 1:**
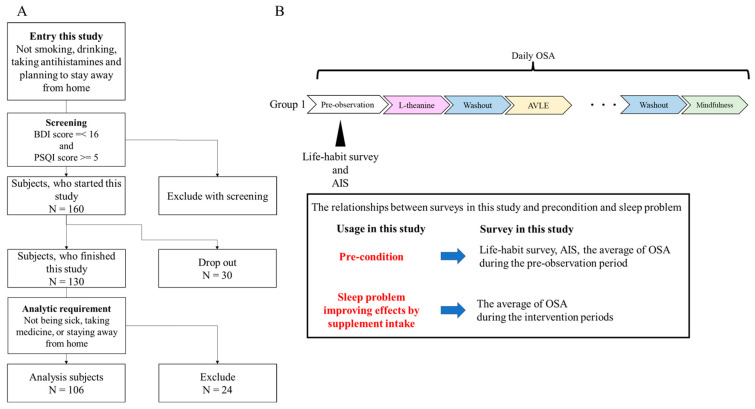
Study design and flowchart of subject screening: (**A**) flowchart of subject screening; (**B**) study design and administration order. BDI is the Beck Depression Inventory, and PSQI is the Pittsburgh Sleep Quality Index.

**Subjects and ethics.** This study was performed according to the Declaration of Helsinki. Before engaging in this study, healthy full-time workers who did not smoke, have a habit of drinking, take antihistamines, or plan to stay outside their home during the pre-observation period and the intervention periods were assessed for eligibility with the Beck Depression Inventory (BDI) and the Pittsburgh Sleep Quality Index (PSQI) [7,8]. In order to select non-depressive subjects with sleep problems, the inclusion criteria were set to under 16 points for the BDI score and more than 5 points for the PSQI score. This study included 160 subjects (Figure 1A). All subjects were divided into 6 groups so that each group’s PSQI and BDI scores were almost the same. The study protocol was approved by the Shiba Palace Clinic Ethics Review Committee (145624-28996). All subjects were informed of the protocol and provided written informed consent prior to the investigation.

**Study design.** An open, randomized, cross-over intervention trial was performed (12 May 2021–26 August 2021). The trial consisted of a pre-observation period of 9 days, followed by six intervention periods of 7 days each and five washout periods of at least 7 days (Figure 1B). The length of the intervention period was decided based on prior research [4,5,6]. In addition, they were limited to 7 days to minimize changes in sleep conditions depending on the day of the week. Intervention and washout periods were set to exclude the 2020 Tokyo Olympics (held in 2021) and Obon holidays (an annual Buddhist event, 6 August 2021–19 August 2021), which may have affected subjects’ sleep. During each of the six intervention periods, each subject took one of the four dietary supplements (l-theanine, GABA, AVLE, or l-serine) or a placebo, or they performed mindfulness. During the pre-observation period, the life-habit survey and the Athens Insomnia Scale (AIS) were administered [9]. During this trial, the Oguri–Shirakawa–Azumi (OSA) sleep questionnaire was administered every morning [10]. The OSA consists of a total score of five factors corresponding to five sleep problems: OSA Factor 1 (sleepiness on rising), OSA Factor 2 (initiation and maintenance of sleep), OSA Factor 3 (frequent dreaming), OSA Factor 4 (refreshing), and OSA Factor 5 (sleep length). We adopted the five OSA factor scores as indices of the corresponding sleep problems. The results of the life-habit survey and AIS and the average of the five OSA factor scores during the pre-observation period were considered as PCs. Subjects answered all assessments on the web.

**Intervention.** During each intervention period of 7 days, each subject was instructed to take either a dietary supplement or placebo or to perform mindfulness. The dietary supplements studied were l-theanine, GABA, AVLE, and l-serine, since these supplements are sold in Japan, reported to have different mechanisms of action and to ameliorate different sleep problems. The dose of each supplement and placebo was determined on the basis of previous reports [4,5,6]: 200 mg/day for l-theanine, 111.1 mg of barley lactic acid fermentation extract/day for GABA, 50 mg/day for AVLE, 300 mg/day for l-serine, and 3300 mg of maltodextrin/day as placebo. All supplements were bulked up to 3300 mg/day with maltodextrin so that the participants did not recognize the ingredient (for the blind intake). These supplements and placebo powders were taken with water within 30–60 min of bedtime. Mindfulness was performed by following a set of mindfulness instructions within 10 min of bedtime [11].

### 2.2. Data and Statistical Analysis

**Analysis subjects and design.** During the study, 30 subjects dropped out. During the intervention periods, 24 subjects were excluded from the data analysis because they experienced one or more of the following potentially confounding conditions for more than 4 days: illness, taking medicine, or staying away from home. A final total of 106 subjects were thus analyzed (Figure 1A and Table 1). We performed two analyses in this study. First, the ability of the individual supplements and mindfulness to improve sleep problems was analyzed (Analysis 1). Second, the relationships between and within supplements, sleep problems, and PCs were examined (Analysis 2).

**Analysis 1.** To confirm the sleep-improving effects of each supplement and mindfulness, the OSA total score and scores for each of the 5 factors during the intervention periods were compared with those of the pre-observation period. It has been well established that sleep problems are often improved by placebo intake [12,13]. Our aim in this study was to extract data regarding PCs and sleep problems to predict the efficacy of dietary sleep-support supplements. Therefore, we compared the improvements in sleep problems before and after the use of the individual supplements, placebo, or mindfulness, and the sleep-improving efficacies were compared among these interventions, rather than comparing the efficacies of supplements to those of the placebo.

Placebo and mindfulness sessions were included as positive controls, in order to examine whether there are specific PCs that might be successfully treated using these interventions. 

**Figure 2:**
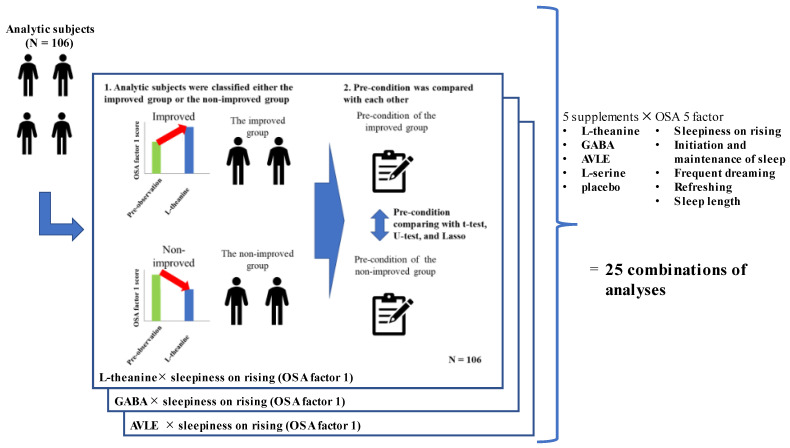
Analysis design.

**Analysis 2.** The relationships between and within supplements, sleep problems, and PCs were examined (Figure 2). For testing 25 combinations (5 OSA factors × 5 supplements), 106 subjects were classified into either the improved group or the non-improved group based on the changes in their OSA score between the intervention and pre-observation period. Subjects whose scores for the 5 OSA factors during the intervention period were higher than those of the pre-observation period were considered to belong to the improved group, and the remaining participants were considered to belong to the non-improved group. For each of the 25 combinations, 31 PCs of the improved group were compared with those of the non-improved group (Table 2). In Analysis 2, placebo and mindfulness interventions were analyzed, but the results of mindfulness are not discussed, since we were unable to control the methods of mindfulness for all participants, and the effects may have varied depending on the methods used.

**Analyses.** All data were calculated as means ± SEMs. In Analysis 1, a paired *t*-test was performed to examine the sleep-improving effects of dietary supplements and mindfulness. In Analysis 2, a univariate analysis (Mann–Whitney U test or Student’s *t*-test) and multivariate analysis (Lasso-regularized general linear model: Lasso) were performed to examine the relationships between and within supplements, sleep problems, and PCs. PCs with a *p* value < 0.05 in the univariate analysis and for which the estimated coefficient in Lasso was not zero were considered to be “significant PCs”. All analyses were performed with Python 3.9.5, scikit-learn 1.0, SciPy 1.8.1, and statsmodels 0.13.2.

## 3. Results

**Analysis 1: Sleep-improving effects of supplements and mindfulness:** Table 3 shows the overall sleep-improving effects of each supplement and mindfulness. Compared with the pre-observation period scores, all the tested supplements, mindfulness, and the placebo significantly improved the OSA total score and scores for OSA Factors 1 and 4. In addition, l-theanine, GABA, l-serine, mindfulness, and the placebo significantly improved the OSA Factor 2 score, and l-theanine significantly improved the OSA Factor 5 score.

**Analysis 2: The relationships between and within supplements, sleep problems, and PCs:** PCs of the improved group were compared with those of the non-improved group for each of the 25 combinations of 5 OSA factors and 5 supplements. Table 4 shows the significant PCs of the improved group for each of the 25 combinations (5 OSA factors × 5 supplements). A summary of the significant PCs for each of the five supplements and placebo is shown in Table 5.

**OSA Factor 1 (sleepiness on rising):** In the l-theanine group, the frequency of dairy product intake, functioning capacity during the day, and OSA Factors 1 and 5 during the pre-observation period were significant PCs. In the GABA group, self-reported bad posture was a significant PC. In the AVLE group, OSA Factor 1 during the pre-observation period was a significant PC. There was no significant PC in the l-serine or placebo group (Table 4 and Supplemental Table S1). 

In regard to OSA Factor 1, l-theanine was effective for the subjects who consumed dairy products frequently and had poor sleep quality (low functioning capacity during the day, sleepiness on rising, and short sleep length). For subjects who had bad posture, GABA was effective. For subjects with sleepiness upon rising, AVLE was effective (Table 5). 

**OSA Factor 2 (initiation and maintenance of sleep):** In the l-theanine group, OSA Factor 2 during the pre-observation period was a significant PC. In the GABA group, OSA Factors 2 and 3 during the pre-observation period were significant PCs. In the AVLE group, the frequency of dairy product intake, and OSA Factors 2 and 3 during the pre-observation period were significant PCs. In the l-serine group, the frequency of bathing and dairy product intake and OSA Factor 2 during the pre-observation period were significant PCs. In the placebo group, the frequency of missed breakfast and OSA Factor 2 during the pre-observation period were significant PCs (Table 4 and Supplemental Table S2).

In regard to OSA Factor 2, l-theanine was effective for the subjects who had a hard time going to sleep and staying asleep. For the subjects who had a hard time going to sleep and staying asleep, and dreamed frequently, GABA was effective. For the subjects who consumed dairy products frequently, had a hard time going to sleep and staying asleep, and dreamed frequently, AVLE was effective. For the subjects who bathed frequently, consumed dairy products frequently, and had a hard time going to sleep and staying asleep, l-serine was effective (Table 5).

**OSA Factor 3 (frequent dreaming):** In the l-theanine group, the frequency of exercising during daylight and weekly physical activities, sleep quality, and OSA Factors 1 and 3 during the pre-observation period were significant PCs. In the GABA group, the frequency of dairy product intake and eating vegetables, and OSA Factor 3 during the pre-observation period were significant PCs. In the AVLE and l-serine groups, OSA Factor 3 during the pre-observation period was a significant PC. In the placebo group, the frequency of taking a bath within 90 min of bedtime and OSA Factor 3 during the pre-observation period were significant PCs (Table 4 and Supplemental Table S3).

With respect to OSA Factor 3, l-theanine was effective for the subjects who exercised frequently and had poor overall sleep quality, especially for those with sleepiness upon rising and who dreamed frequently. For the subjects who consumed dairy products frequently, ate vegetables frequently, and dreamed frequently, GABA was effective. For the subjects who dreamed frequently, AVLE and l-serine were effective (Table 5).

**OSA Factor 4 (refreshing):** In the l-theanine group, the frequency of smartphone or computer usage, bathing, and dairy product intake, and OSA Factors 2, 3, and 4 during the pre-observation period were significant PCs. In the GABA group, the frequency of smartphone usage before bedtime, early awakening, and OSA Factor 4 during the pre-observation period were significant PCs. In the AVLE, l-serine, and placebo groups, OSA Factor 4 during the pre-observation period was a significant PC (Table 4 and Supplemental Table S4). 

In regard to OSA Factor 4, l-theanine was effective for the subjects who had good life habits (used a smartphone or computer infrequently, bathed frequently, and consumed dairy products frequently) and had poor sleep quality (had a hard time going to sleep and staying asleep, dreamed frequently, and were not refreshed). For the subjects who did not use a smartphone at night frequently, did not wake up early frequently, and were not refreshed, GABA was effective. For the subjects who were not refreshed, AVLE and l-serine were effective (Table 5).

**OSA Factor 5 (sleep length):** In the l-theanine group, the frequency of smartphone or computer usage and missing breakfast, and OSA Factor 5 during the pre-observation period were significant PCs. In the GABA group, the frequency of smartphone usage before bedtime and OSA Factor 5 during the pre-observation period were significant PCs. In the AVLE group, the frequency of having meals at irregular times, awakenings during the night, sleep quality, and OSA Factor 5 during the pre-observation period were significant PCs. In the l-serine and placebo groups, OSA Factor 5 during the pre-observation period was a significant PC (Table 4 and Supplemental Table S5).

In terms of OSA Factor 5, l-theanine was effective for the subjects who did not use a smartphone or computer frequently, did not eat breakfast every day, and slept for a short period of time. For the subjects who did not use a smartphone at night frequently and slept for a short period of time, GABA was effective. For the subjects who had meals at irregular times, had better overall sleep quality, and slept for a short period of time, AVLE was effective. For the subjects who slept for a short period of time, l-serine was effective (Table 5).

## 4. Discussion

In this study, Analyses 1 and 2 were performed to examine the relationships between and within dietary supplements, sleep problems, and PCs. In Analysis 1, compared with OSA scores in the pre-observation period, l-theanine, GABA, AVLE, l-serine, mindfulness, and the placebo all significantly improved the OSA total score and the scores of OSA Factors 1, 2, 4, and 5. In Analysis 2, the PCs of the improved group were compared with those of the non-improved group for each of the 25 combinations of the 5 OSA factors and 5 supplements (l-theanine, GABA, AVLE, l-serine, and placebo). As a result, for each combination, the significant PCs were different.

Dietary supplements may be superior to pharmacotherapy in their safety profiles and convenience to use, and many studies have been focused on assessing improvement in sleep problems through supplement intake compared with placebo intake [4,5,6]. However, these studies did not adequately account for the potential sleep-improving effects of placebo, or for the effects that the unique circumstances and conditions of subjects had on supplement’s efficacy. In particular, to the best of our knowledge, there has been only one study focused on individual differences in the effects of dietary supplements [6]. To overcome these limitations, we herein examined the improvement in sleep problems by comparing the OSA scores before and after various interventions, and the relationships between and within dietary supplements, sleep problems, and PCs. 

Our results showed that each of the dietary supplements, the placebo, and mindfulness all significantly improved the OSA total score and the scores of OSA Factors 1, 2, 4, and 5. It was also notable that all the tested supplements had sleep-improving efficacies better or similar to those of the placebo and mindfulness. Most importantly, we found that PCs were more helpful for predicting the efficacy of dietary supplements than for predicting the efficacy of the placebo and mindfulness and that the dietary supplement with the greatest efficacy was dependent on a user’s PCs and sleep problems. 

These results suggest the possibility of personalizing supplementation based on a user’s PCs, rather than merely selecting a dietary supplement based on the sleep-improving effect of supplement intake. Therefore, this study confirms the efficacy of dietary sleep-support supplements and further extends prior research by clarifying the significant PCs that impact personalized supplementation. As a result, the possibility of implementing personalized supplementation based on significant PCs was suggested. For example, a high frequency of eating vegetables was a PC that was only observed in the subjects whose OSA Factor 3 (frequent dreaming) was improved via GABA intake (see Table 5 and the OSA Factor 3 results in Analysis 2). This result suggests that GABA would be more effective for people who eat vegetables frequently. However, the mechanism underlying the relationship between the sleep-improving effects of GABA, the habit of eating vegetables, and the sleep problem of dreaming frequently is uncertain. Previous reports show that dietary habits can modify gene expressions and the microbiome through the consumption of certain functional ingredients. For example, it has been reported that the ethyl acetate extract from tomato juice inhibits CYP3A4 activity [14], and polyphenols, vitamins, minerals, and dietary fiber modulate the health-beneficial gut microbes [15,16]. The modification in genes and the microbiome may affect the ADME of each supplement, and therefore the sleep-improving effects of each supplement may not be uniform. In the case of GABA intake, some functional ingredients contained in some vegetables may affect the GABA-specific ADME and enhance the sleep-improving effect of GABA. As another example, we found that each of the supplements studied improved the sleep problems of individuals who consumed dairy products (Table 6). Previous studies have reported that sleep problems were improved by consuming certain functional ingredients in dairy products [17,18,19,20]. For example, α-lactalbumin reduces sleepiness by increasing plasma tryptophan [19]. In our study, in the case of placebo intake, there was no difference in the consumption frequency of dairy products between the improved group and the non-improved group. This may suggest that the intake of dairy products specifically enhances the sleep-improving effects of dietary supplements.

## 5. Prospects

This study suggests the possibility of life-habit recommendations to enhance the efficacy of dietary supplements based on supplements and sleep problems. For example, if l-serine is taken to improve going to and maintaining sleep (OSA Factor 2), bathing frequently can be recommended (as described in the OSA Factor 2 results for Analysis 2 and shown in Table 5). However, this study was an observational study for examining the amelioration of sleep problems via the combination of dietary supplements and life-habit interventions. Additional interventional studies will be needed to examine the degree to which life-habit recommendations can enhance the sleep-improving effects of dietary supplements.

## 6. Limitations

In this study, the relationships between and within the efficacy of dietary supplements, sleep problems, and PCs were explored. We succeeded in identifying the PCs and sleep problems that influence the efficacy of dietary sleep-support supplements. Individual patients should benefit from the use of our criteria to select dietary sleep-support supplements. However, little information is currently available in regard to how individual biological and genetic characteristics affect a supplement’s efficacy. It is therefore possible that more significant PCs will be identified in the future, and these will also be incorporated to personalize supplementation more precisely based on personal life habits, sleep conditions, sleep problems, and biological markers to support the sleep of specific individuals. In parallel, the mechanisms involved in the relationships between and within PCs, sleep problems, and the efficacy of dietary sleep-support supplements should be studied. However, it is not practical to reveal all mechanisms; therefore, to reveal the interesting interactions and mechanisms, in vitro and in vivo studies should be considered. In particular, enhancing supplement efficacy by taking dairy products is a novel approach with broad effects, so it is important to examine its mechanisms to evaluate the benefit and risks of this method.

## 7. Conclusions

In this research, the relationships between and within the PCs and estimations of improvement in sleep problems through dietary sleep-support supplements were revealed. The results suggest the possibility of implementing personalized sleep-improving supplementation based on personal life habits, sleep conditions, and sleep problems.

## Figures and Tables

**Table 1:** Demographic information.

	Total (N = 106)
**Demographic Information**	
Age, Mean (±SEM)	45.5 (±0.7)
Male, Num	38
Female, Num	68
BDI, Mean (±SEM)	5.1 (±0.5)
PSQI, Mean (±SEM)	7.9 (±0.2)

Data are shown as means ± SEMs. BDI is the Beck Depression Inventory, and PSQI is the Pittsburgh Sleep Quality Index.

**Table 2:** List of PCs.

Pre-Condition	
Life Habit	Sleep Condition
Number of steps a day ^a^	Sleep induction ^e^
Sitting for more than one hour at least three times a day ^b^	Awakenings during the night ^e^
Bad posture ^b^	Final awakening ^e^
Using a smartphone or computer frequently ^b^	Total sleep duration ^e^
Using a smartphone at least 3 times a week before bedtime ^b^	Sleep quality ^e^
Bathing in a bath ^b^	Well-being during the day ^e^
Exercise during daylight ^b^	Functioning capacity during the day ^e^
Taking a bath within 90 min of bedtime ^b^	Sleepiness during the day ^e^
Stretching at least twice a week ^b^	**OSA** Factor 1 during the pre-observation period (Sleepiness on rising) ^f^
Eating fermented food at least 3 times a week ^b^	**OSA** Factor 2 during the pre-observation period (Initiation and maintenance of sleep) ^f^
Eating dairy products at least 3 times a week ^b^	**OSA** Factor 3 during the pre-observation period (Frequent dreaming) ^f^
Not drinking caffeine after 3 PM ^b^	**OSA** Factor 4 during the pre-observation period (Refreshing) ^f^
Meals at irregular times ^b^	**OSA** Factor 5 during the pre-observation period (Sleep length) ^f^
Missed breakfast at least 3 times a week ^b^	
Meals within 2 h of bedtime at least 3 times a week ^b^	
Eating vegetables at least 3 times a day ^b^	
Weekly physical activities ^c^	
Drinking ^d^	

^a.^ <2000 steps: 1 point; 2000–4000 steps: 2 points; 4000–6000 steps: 3 points; 6000–8000 steps: 5 points; 10,000–12,000 steps: 6 points; >12,000 steps: 7 points. ^b.^ Yes: 1 point; No: 2 points. ^c.^ 0 min: 1 point; <30 min: 2 points; 30–59 min: 3 points; ≥60 min: 4 points. ^d.^ <1 unit: 1 point; 1–2 units: 2 points; 2–3 units: 3 points; >3 units: 4 points (1 unit = 500 mL beer, 180 mL sake, 30 mL whiskey, or 180 mL wine). ^e.^ Athens Insomnia Scale. ^f.^ The average of the Oguri–Shirakawa–Azumi (OSA) sleep questionnaire 5 factors’ scores during the pre-observation period.

**Table 3:** Sleep-improving effects of supplements and mindfulness.

	Pre-Observation		l-Theanine			GABA			AVLE			l-Serine			Mindfulness			Placebo		
	Mean	SEM	Mean	SEM	*p* Value (*t*-Test)	Mean	SEM	*p* Value (*t*-Test)	Mean	SEM	*p* Value (*t*-Test)	Mean	SEM	*p* Value (*t*-Test)	Mean	SEM	*p* Value (*t*-Test)	Mean	SEM	*p* Value (*t*-Test)
OSA total score	86.29	(±1.69)	92.48	(±1.82)	*p* < 0.001 ***	90.55	(±1.87)	0.007 **	90.09	(±1.83)	0.012 *	90.06	(±1.92)	0.012 *	89.63	(±1.71)	0.030 *	91.17	(±1.86)	0.003 **
OSA Factor 1 Sleepiness on rising	16.93	(±0.44)	18.67	(±0.51)	*p* < 0.001 ***	18.25	(±0.52)	0.006 **	18.43	(±0.50)	*p* < 0.001 ***	18.59	(±0.53)	*p* < 0.001 ***	17.98	(±0.50)	0.019 *	18.56	(±0.53)	0.001 **
OSA Factor 2 Initiation and maintenance of sleep	15.08	(±0.41)	16.68	(±0.46)	*p* < 0.001 ***	16.42	(±0.43)	*p* < 0.001 ***	15.73	(±0.41)	0.097	15.95	(±0.42)	0.018 *	16.24	(±0.43)	0.004 **	16.40	(±0.43)	0.001 **
OSA Factor 3 Frequent dreaming	20.33	(±0.66)	20.41	(±0.64)	0.866	20.21	(±0.66)	0.794	19.81	(±0.67)	0.281	19.72	(±0.66)	0.199	20.27	(±0.65)	0.907	20.22	(±0.65)	0.814
OSA Factor 4 Refreshing	16.19	(±0.46)	18.03	(±0.47)	*p* < 0.001 ***	17.32	(±0.48)	0.015 *	17.85	(±0.46)	*p* < 0.001 ***	17.50	(±0.49)	0.002 **	17.18	(±0.43)	0.019 *	17.55	(±0.48)	0.008 **
OSA Factor 5 Sleep length	17.78	(±0.48)	18.69	(±0.49)	0.030 *	18.35	(±0.49)	0.18	18.28	(±0.50)	0.169	18.31	(±0.51)	0.184	17.96	(±0.50)	0.656	18.45	(±0.51)	0.112

Data are shown as means ± SEMs. * *p* < 0.05, ** *p* < 0.01, *** *p* < 0.001 using paired *t*-test.

**Table 4:** The significant PCs of the improved group.

OSA Factor 1			
Supplement	Pre-Condition	*p* Value (U/*t* Test)	Coefficient
l-Theanine	Eating dairy products at least 3 times a week	0.013 *	−0.087
	Functioning capacity during the day	0.035 *	0.031
	OSA Factor 1 during the pre-observation period	0.001 **	−0.097
	OSA Factor 5 during the pre-observation period	0.016 *	−0.041
GABA	Bad posture	0.033 *	−0.042
AVLE	OSA Factor 1 during the pre-observation period	0.014 *	−0.055
l-Serine	No feature		
Mindfulness	Eating dairy products at least 3 times a week	0.013 *	−0.069
	Taking a bath within 90 min of bedtime	0.032 *	0.038
	OSA Factor 1 during the pre-observation period	0.049 *	−0.023
	OSA Factor 4 during the pre-observation period	0.045 *	−0.0058
Placebo	No feature		
**OSA Factor 2**			
**Supplement**	**Pre-condition**	***p* value** **(U/*t* test)**	**coefficient**
l-Theanine	OSA Factor 2 during the pre-observation period	*p* < 0.001 ***	−0.071
GABA	OSA Factor 2 during the pre-observation period	*p* < 0.001 ***	−0.1
	OSA Factor 3 during the pre-observation period	0.0011 **	−0.059
AVLE	Eating dairy products at least 3 times a week	0.0092 **	−0.084
	OSA Factor 2 during the pre-observation period	0.0010 **	−0.119
	OSA Factor 3 during the pre-observation period	0.014 *	−0.022
l-Serine	Bathing in a bath	0.046 *	−0.061
	Eating dairy products at least 3 times a week	0.0033 **	−0.094
	OSA Factor 2 during the pre-observation period	*p* < 0.001 ***	−0.178
Mindfulness	Using your smartphone or computer frequently	0.018 *	0.076
	OSA Factor 2 during the pre-observation period	*p* < 0.001 ***	−0.16
Placebo	Missed breakfast at least 3 times a week	0.022 *	0.019
	OSA Factor 2 during the pre-observation period	0.0011 **	−0.067
**OSA Factor 3**			
**Supplement**	**Pre-condition**	***p* value** **(U/*t* test)**	**coefficient**
l-Theanine	Exercise during daylight	0.004 **	−0.064
	Weekly physical activities	0.002 **	0.086
	Sleep quality	0.047 *	0.014
	OSA Factor 1 during the pre-observation period	0.040 *	−0.012
	OSA Factor 3 during the pre-observation period	*p* < 0.001 ***	−0.138
GABA	Eating dairy products at least 3 times a week	0.0053 **	−0.085
	Eating vegetables at least 3 times a day	0.0091 **	−0.08
	OSA Factor 3 during the pre-observation period	0.024 *	−0.057
AVLE	OSA Factor 3 during the pre-observation period	0.0015 **	−0.065
l-Serine	OSA Factor 3 during the pre-observation period	*p* < 0.001 ***	−0.086
Mindfulness	Stretching at least twice a week	0.017 *	0.074
	Sleep quality	0.004 **	0.065
	OSA Factor 3 during the pre-observation period	*p* < 0.001 ***	−0.108
Placebo	Taking a bath within 90 min of bedtime	0.032 *	−0.024
	OSA Factor 3 during the pre-observation period	*p* < 0.001 ***	−0.089
**OSA Factor 4**			
**Supplement**	**Pre-condition**	***p* value** **(U/*t* test)**	**coefficient**
l-Theanine	Using your smartphone or computer frequently	*p* < 0.001 ***	0.111
	Bathing in a bath	0.046 *	−0.037
	Eating dairy products at least 3 times a week	0.012 *	−0.095
	OSA Factor 2 during the pre-observation period	0.012 *	−0.012
	OSA Factor 3 during the pre-observation period	0.019 *	−0.026
	OSA Factor 4 during the pre-observation period	*p* < 0.001 ***	−0.097
GABA	Using your smartphone at least 3 times a week before bedtime	0.030 *	−0.029
	Final awakening	0.024 *	−0.041
	OSA Factor 4 during the pre-observation period	0.0034 **	-0.069
AVLE	OSA Factor 4 during the pre-observation period	0.0032 **	−0.051
l-Serine	OSA Factor 4 during the pre-observation period	*p* < 0.001 ***	−0.037
Mindfulness	Taking a bath within 90 min of bedtime	0.005 **	0.044
	Eating vegetables at least 3 times a day	0.026 *	−0.039
	OSA Factor 2 during the pre-observation period	0.005 **	−0.023
	OSA Factor 4 during the pre-observation period	*p* < 0.001 ***	−0.107
Placebo	OSA Factor 4 during the pre-observation period	*p* < 0.001 ***	−0.098
**OSA Factor 5**			
**Supplement**	**Pre-condition**	***p* value** **(U/*t* test)**	**coefficient**
l-Theanine	Using your smartphone or computer frequently	0.0096 **	0.078
	Missed breakfast at least 3 times a week	0.0054 **	−0.06
	OSA Factor 5 during the pre-observation period	0.0011 **	−0.091
GABA	Using your smartphone at least 3 times a week before bedtime	0.042 *	−0.059
	OSA Factor 5 during the pre-observation period	*p* < 0.001 ***	−0.148
AVLE	Meals at irregular times	0.041 *	−0.068
	Awakenings during the night	0.021 *	−0.11
	Sleep quality	0.034 *	−0.056
	OSA Factor 5 during the pre-observation period	0.0045 *	−0.115
l-Serine	OSA Factor 5 during the pre-observation period	0.013 *	−0.008
Mindfulness	Total sleep duration	0.049 *	−0.024
	OSA Factor 5 before administration	*p* < 0.001 ***	−0.138
Placebo	OSA Factor 5 during the pre-observation period	*p* < 0.001 ***	−0.102

Mann–Whitney U test and Student’s *t*-test were performed to calculate *p* value (U/*t* test). Coefficients were calculated using Lasso regression analysis. * *p* < 0.05, ** *p* < 0.01, *** *p* < 0.001.

**Table 5:** Summary of significant PCs of supplements.

	OSA Factor 1 Sleepiness on Rising	OSA Factor 2 Initiation and Maintenance of Sleep	OSA Factor 3 Frequent Dreaming	OSA Factor 4 Refreshing	OSA Factor 5 Sleep Length
l-Theanine	Eating dairy products frequently Low functioning capacity during the day Sleepiness on rising Short sleep length	Having a hard time going to sleep and staying asleep	Exercising frequently Bad overall sleep quality Sleepiness on rising Dreaming frequently	Using a smartphone/computer infrequently Bathing frequently Eating dairy products frequently Having a hard time going to sleep and staying asleep Dreaming frequently Not refreshed	Using a smartphone/computer infrequently Missed breakfast frequently Short sleep length
GABA	Bad posture	Having a hard time going to sleep and staying asleep Dreaming frequently	Eating dairy products frequently Eating vegetables frequently Dreaming frequently	Using a smartphone at midnight infrequently Early awakening infrequently Not refreshed	Using a smartphone at midnight infrequently Short sleep length
AVLE	Sleepiness on rising	Eating dairy products frequently Having a hard time going to sleep and staying asleep Dreaming frequently	Dreaming frequently	Not refreshed	Meals at irregular times Awakening during the night infrequently Good sleep quality Short sleep length
l-Serine	-	Bathing frequently Eating dairy products frequently Having a hard time going to sleep and staying asleep	Dreaming frequently	Not refreshed	Short sleep length
Placebo	-	Missed breakfast infrequently Having a hard time going to sleep and staying asleep	Taking a bath within 90 min of bedtime Dreaming frequently	Not refreshed	Short sleep length

**Table 6:** Effect of dairy products.

	l-Theanine	GABA	AVLE	l-Serine	Placebo
OSA Factor 1 Sleepiness on rising	**+++**	**+**	**+**	−	−
OSA Factor 2 Initiation and maintenance of sleep	−	**+**	**+++**	**+++**	−
OSA Factor 3 Frequent dreaming	−	**+++**	**+**	−	−
OSA Factor 4 Refreshing	**+++**	−	−	**+**	−
OSA Factor 5 Sleep length	**+**	**+**	**+**	−	−

+++: *p* < 0.05 in univariate analysis (Mann–Whitney U test), and coefficient is not zero in multivariate analysis (Lasso regression analysis). +: 0.05 ≤ *p* < 0.1 in univariate analysis (Mann–Whitney U test) or coefficient is not zero in multivariate analysis (Lasso regression analysis). −: *p* ≥ 0.1 in univariate analysis (Mann–Whitney U test) or coefficient is zero in multivariate analysis (Lasso regression analysis).

## Data Availability

Data sharing is not applicable to this article.

## Supplementary Materials

The following supporting information can be downloaded at: https://www.mdpi.com/article/10.3390/nu15102377/s1. Table S1: Statistical analysis result of OSA Factor 1 (sleepiness on rising), Table S2: Statistical analysis result of OSA Factor 2 (initiation and maintenance of sleep), Table S3: Statistical analysis result of OSA Factor 3 (frequent dreaming), Table S4: Statistical analysis result of OSA Factor 4 (refreshing), Table S5: Statistical analysis result of OSA Factor 5 (sleep length).
